# Solid-State Processing of CoCrMoNbTi High-Entropy Alloy for Biomedical Applications

**DOI:** 10.3390/ma16196520

**Published:** 2023-09-30

**Authors:** Alina Elena Bololoi, Laura Elena Geambazu, Iulian Vasile Antoniac, Robert Viorel Bololoi, Ciprian Alexandru Manea, Vasile Dănuţ Cojocaru, Delia Pătroi

**Affiliations:** 1 Materials Science and Engineering Faculty, National University of Science and Technology Politehnica Bucharest, Splaiul Independentei 313, 060042 Bucharest, Romania; alina_elena.boarna@upb.ro (A.E.B.); antoniac.iulian@gmail.com (I.V.A.); robert.bololoi@upb.ro (R.V.B.); ciprian.manea@icpe-ca.ro (C.A.M.); dan.cojocaru@upb.ro (V.D.C.); 2 National Institute for R&D in Electrical Engineering ICPE-CA Bucharest, Splaiul Unirii 313, 030138 Bucharest, Romania; delia.patroi@icpe-ca.ro

**Keywords:** CoCrMoNbTi, high-entropy alloys, mechanical alloying, biomaterial, biomedical

## Abstract

High-entropy alloys (HEAs) gained interest in the field of biomedical applications due to their unique effects and to the combination of the properties of the constituent elements. In addition to the required property of biocompatibility, other requirements include properties such as mechanical resistance, bioactivity, sterility, stability, cost effectiveness, etc. For this paper, a biocompatible high-entropy alloy, defined as bio-HEA by the literature, can be considered as an alternative to the market-available materials due to their superior properties. According to the calculation of the valence electron concentration, a majority of body-centered cubic (BCC) phases were expected, resulting in properties such as high strength and plasticity for the studied alloy, confirmed by the XRD analysis. The tetragonal (TVC) phase was also identified, indicating that the presence of face-centered cubic (FCC) phases in the alloyed materials resulted in high ductility. Microstructural and compositional analyses revealed refined and uniform metallic powder particles, with a homogeneous distribution of the elemental particles observed from the mapping analyses, indicating that alloying had occurred. The technological characterization of the high-entropy alloy-elaborated powder revealed the particle dimension reduction due to the welding and fracturing process that occurs during mechanical alloying, with a calculated average particle size of 45.12 µm.

## 1. Introduction

High-entropy alloys (HEA) are formed from five or more principal elements, in 5 to 35 at% each according to the literature [1,2]. These alloys are known to exhibit unique and superior properties as high corrosion, erosion and wear resistance, but also excellent mechanical behavior [3,4], besides their unique effects such as the high-entropy effect, slow diffusion effect, lattice disorder effect and cocktails effect [1]. Due to these specific effects, high-entropy alloys exhibit unique microstructural features [5,6], resulting in considerable interest in the recent research [7,8,9].

In order to determine the crystalline structures such as hexagonal closed packed (HCP), FCC or BCC that usually form in HEA, valence electron concentration (VEC) determination is required due to the random solid solutions forming, which are caused by the high-entropy effect [3]. According to the theory depicted in [6], for a calculated value of 6.8 to 8, a mixture of BCC and FCC phases will be present. BCC phases will be present when the calculated value is lower than 6.8, indicating relatively high fracture strength, but also low ductility. Furthermore, FCC phases will be present if the calculated value is greater than 8, indicating low strength but excellent ductility in the final material.

Based on research that presented the superior properties and application possibilities [5,10,11,12], the high-entropy alloys gained interest in the biomedical field applications due to their tailored properties including mechanical resistance and biocompatibility [3].

When discussing medical implants, high biocompatibility and excellent mechanical properties are required. The high-entropy alloys present a high interest in the medical field due to their unique composition, design freedom, tailored properties and even potential shape memory capability [13], besides homogeneous microstructure, corrosion and wear resistance [14,15,16,17].

In a paper published by Yang et.al. [18], TiZrHfNbTa HEA in the equiatomic ratio produced with the arc-melting method presented bio-corrosion resistance, cell adhesion, proliferation and viability when tested in MC3T3-E1 cell culture. The alloy exhibited spontaneous passivation on the surface when exposed to the media, indicating in vitro biocompatibility. Hua et.al. [19] studied the TiZrNbTaMo system, with the Ti concentration variation (molar ratio of 0.5, 1, 1.5, 2) produced by being arc melted and remelted for at least 5 times, which presented a dendritic structure composed of BCC phases with superior dry and wet wear resistance when compared to the Ti6Al4V alloy. When the Ti concentration was reduced to a molar ratio of 0.5, it was observed that the alloy exhibited an improved corrosion and wear resistance.

According to a paper published by Liu et.al [13], a potential risk of corrosion was observed for the metallic endoprosthesis implanted in the human body, which generally leads to issues such as decreased performance and implant failure. When exposing biocompatible high-entropy alloys to a corrosive environment, a passivation oxide film is formed on the surface, reducing the corrosion degree and the risk of material failure, leading to the possibility of producing bio-HEA for applications in the biomedical domain.

Besides the arc-melting technique used to produce high-entropy alloys, stationary friction processing (SFP) [20], powder metallurgy (PM) [21] and selective laser melting [22,23] were taken into consideration in order to increase the elemental homogenization and the alloying degree of these materials, leading to results such as bio-corrosion resistance [20], high resistance to dissolution in acids [21,24], osteoblast adhesion and refined microstructure [3,23].

The work from this paper was focused on solid-state processing of CoCrMoNbTi HEA using mechanical alloying in a planetary ball mill with the goal of producing a high alloying degree and homogeneity and to further process it into a final product. Another advantage brought by using solid-state processing, such as undesirable dendritic structures, or segregations, which are generally specific to the liquid state processing method, are avoided in the materials elaboration.

The elemental materials were down-selected based on their biocompatibility properties but also corrosion and wear resistance when used in the following medical field applications.

Molybdenum was studied by C. Redlich et.al. [25] for its potential as a biodegradable implant material that exhibits higher strength when compared to iron-based materials, with a gradual dissolution that could form soluble corrosion products and low toxicity on cell studies. Co-Cr alloys have been used in medical applications as materials for dental and medical devices due to their high corrosion and wear resistance and excellent mechanical properties, besides biocompatibility, according to the literature [26,27,28].

Titanium alloys have been utilized in medical applications for dental and orthopedics implants, where commercially pure titanium and Ti6Al4V alloys have been reported to exhibit properties such as high elastic modulus (110 GPa) and higher elastic stiffness when compared to the cortical bone (15–30 GPa) [29,30,31]. Due to the risks of using Al and V, which release toxic metal when in contact with the human body media, after long-term implantation [32], research studies have been focused on developing new Nb-Ti-Zr alloys [31,33], Nb-Ta-Ti-Zr alloys [34] and Mo-Nb-Ti-Zr alloys [35]. O. Mishchenko et.al [31] highlighted in their research that when tested on cell culture, the Nb-Ti-Zr alloy produced with vacuum arc melting demonstrated full biocompatibility, and T. Zhang et.al [33] observed properties such as excellent chemical stability and micropores that enhance both adhesion and proliferation of osteoblasts when the alloy was produced via pressing and sintering of the mixed elemental powders.

The down-selection of the mechanical alloying parameters for this work was based on recent research in the field of corrosion/erosion resistant [4,36] high-entropy alloys, but also by evaluating the alloying degree during solid-state processing in order to achieve the CoCrMoNbTi HEA powder.

The importance of this work is mainly represented by the need of producing materials with superior properties for the biomedical domain which could reduce risks such as corrosion, erosion or failure of the used biomedical equipment. Developing CoCrMoNbTi HEA using solid-state processing with a high homogeneity degree and high alloying degree represents the initial steps toward developing coatings for surgical tools using various deposition methods.

## 2. Materials and Methods

For this research, high-purity (approx. 99%) metallic powders of cobalt, chromium, molybdenum, niobium and titanium (Alfa Aesar, Haverhill, MA, USA), as constituent elemental materials, were used in equiatomic ratio in order to obtain CoCrMoNbTi high-entropy alloy using solid-state processing. Powder manipulation during the experimentation was performed in a glovebox equipped with an oxygen monitor in argon atmosphere, reducing the oxidation that might occur during the process. The nominal composition of the elemental raw materials employed in this work is reported in Table 1.

The valence electron concentration, which has been reported in the literature [3,6] as an indicator of the phase stability in high-entropy alloys, was calculated using Equation (1).

(1) $$\mathrm{VEC}=\sum_{i=1}^{n}{VEC\;}_{i}\cdot c_{i}$$

Elemental metallic powder blend was obtained by mixing the raw materials for 10 min in the planetary ball mill (Fritsch–Pulverisette 6^®^, Idar-Oberstein, Germany) used for the alloying process, and with no process control agent a sample was collected and analyzed. The purpose of the mixing step was to observe the evolution of the alloying degree starting with the raw powders in their initial shape and size. The powders were mixed at 150 RPM with no milling media in a stainless-steel vial.

For the mechanical alloying process, a planetary ball mono-mill (Fritsch–Pulverisette 6^®^, Idar-Oberstein, Germany) with a stainless-steel vial and milling balls (dia. 15 mm and 20 mm) used. The alloying degree evolution was monitored by collecting samples from the mixture once every 6 h. To reduce the contamination and oxidation degree during alloying, the vial was flooded with high-purity argon. Wet milling was considered due to the improvement of the alloying efficiency and decreasing tendency of the metallic powder to adhere to the milling media, with 2 wt% N-heptane as a process control agent (PCA). The selected milling process parameters used were as follows: grinding speed of 300 RPM with 10:1 BPR (ball to powder ratio) for a period of 30 h to obtain a high alloying degree. The final alloy was sieved with vibratory sieve shaker (Fritsch–Analysette 3^®^ SPARTAN, Idar-Oberstein, Germany) equipment for measuring the quantitative size distribution of the particles with 20 μm, 32 μm, 45 μm, 56 μm, 63 μm, 80 μm, 100 μm, 125 μm, 140 μm and 160 μm aperture sieves.

The technological characterization of the CoCrMoNbTi HEA produced with mechanical alloying was achieved by measuring flow rate and slope angle with calibrated Hall flow meter equipment and free flow and tap density with a graduated cylinder.

In order to determine the influence of mechanical alloying on the alloy in the form of metallic powder, the tests were also carried out on the elemental metallic powder materials and compared. The microscopical SEM, EDS and Mapping analyses were performed with Tescan Vega II-XMU SEM equipment (Tescan Group, Kohoutovice, Czech Republic) coupled with a Bruker xFlash 6/30 EDS detector (Bruker, Cordoba, Argentina).

X-ray diffraction analysis was carried out using the D8-Discover diffractometer, Bruker, Germany, with Cu primary radiation (λ = 1.540598 Å), Goebel mirror and 1D LynxEye detector (Bruker, Cordoba, Argentina) on the secondary side. The diffractograms were obtained with an angular increment of 0.04°, at a scanning speed of 1 s/step in the Physical-Chemical Testing Laboratory, LI-MAT, within the National Research and Development Institute for Electrical Engineering ICPE-CA, Bucharest. The identification of the crystallographic phases was carried out using the ICDD PDF 2 Release 2022 database and the structures were found to be similar to the indexed files PDF 00-035-0789 for niobium, PDF 03-065-7442 for molybdenum, PDF 00-006-0694 for chromium, PDF 01-087-4196 for titanium and PDF 01-065-9205 for Co0.86Nb0.14.

## 3. Results and Discussion

**Figure 1:**
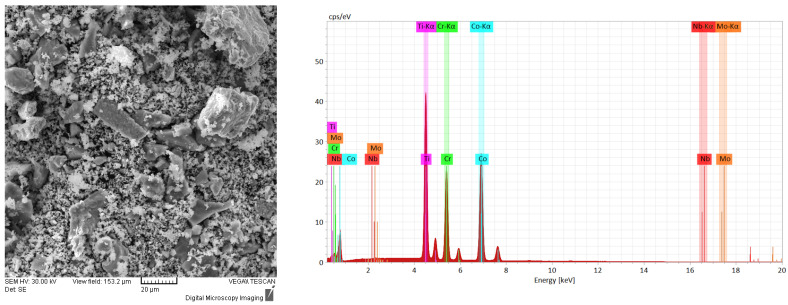
Results of the SEM images and EDS analyses for the mixed sample of the CoCrMoNbTi mixture.

**Figure 2:**
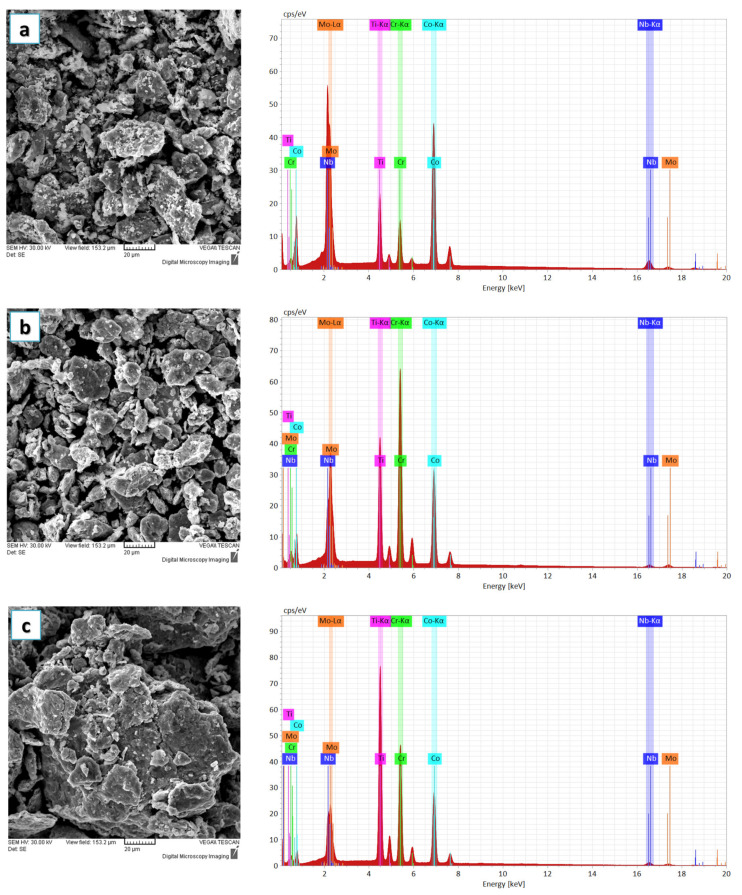

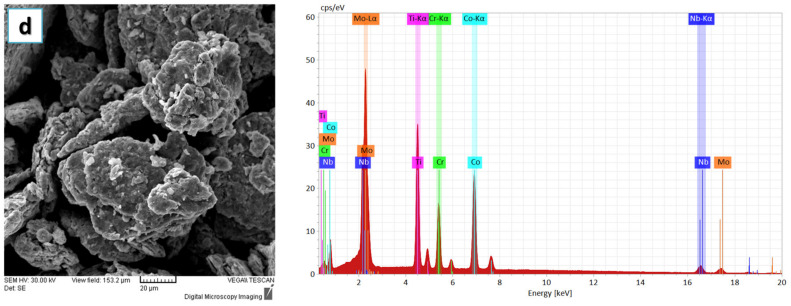
Results of the SEM images and EDS analyses for samples collected after (**a**) 6 h, (**b**) 12 h, (**c**) 18 h and (**d**) 24 h of mechanical alloying of the CoCrMoNbTi mixture.

**Figure 3:**
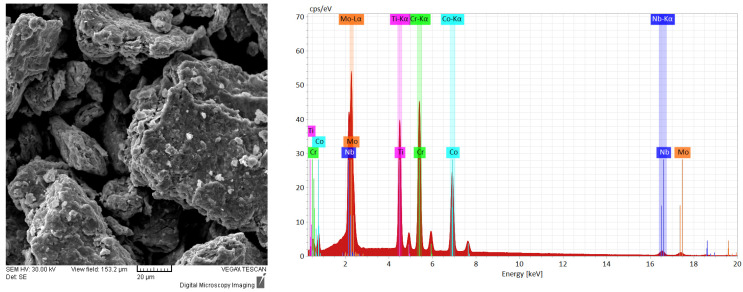
Results of the SEM images and EDS analyses for the sample collected after 30 h of mechanical alloying of the CoCrMoNbTi mixture.

The results of the SEM and EDS analyses obtained for the metallic powder during the alloying process are presented in Figure 1, Figure 2 and Figure 3. As it can be observed in Figure 1, the mixed sample presents the elemental material particles with different geometric shapes and dimensions. It was observed from the EDS analyses that no contamination was present from the incipient states of the process and that the composition was confirmed.

The identified peaks confirm the presence of the pure component materials. In order to have a quantitative overview on the chemical composition of the analyzed samples, the resulting data are correspondingly presented in weight percentages. The literature [37] presents that, when mixing the metallic powder for 6 h, the powder morphology was suitable for laser cladding due to the uniform shape and size observed from the SEM analyses; although, in this case, the particles presented a more spherical shape, which was probably influenced by the alloying media and parameters.

Figure 2a–d present the evolution of the alloying degree and morphology changes that took place with the extension of the material milling time for CoCrMoNbTi HEA. Particle agglomeration and particle shape modifications were observed after 6 h milling, indicating a high milling efficiency due to the use of N-Heptane in the mixing and the mechanical alloying during the incipient phase. No compositional modifications or oxidation were present during the process. As N-Heptane traces were not identified in the composition, this indicates that it evaporated during the process and that this PCA has a boiling point of 98.38 °C.

In Figure 3, the alloyed powder material was microstructurally analyzed. The results presented an improved agglomeration of the particles, which presented a polygonal shape and uniform color throughout the analyzed sample, indicating that the CoCrMoNbTi HEA was obtained after 30 h of milling in a planetary ball mill.

**Figure 4:**
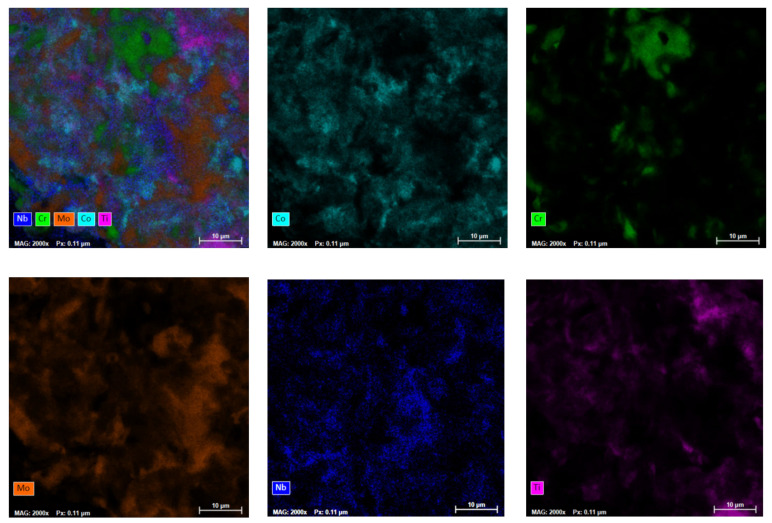
Mapping analyses for CoCrNbMoTi high-entropy alloy obtained after 30 h of milling.

In order to observe the elemental distribution of the elements in the powder materials, mapping analyses were performed, and the results are presented in Figure 4. Mapping analyses were performed on the area microstructurally analyzed and shown in Figure 3.

It was observed that the agglomerated particles presented the elemental metallic materials, but a good distribution of the constituent elements was also observed throughout the analyzed sample.

**Figure 5:**
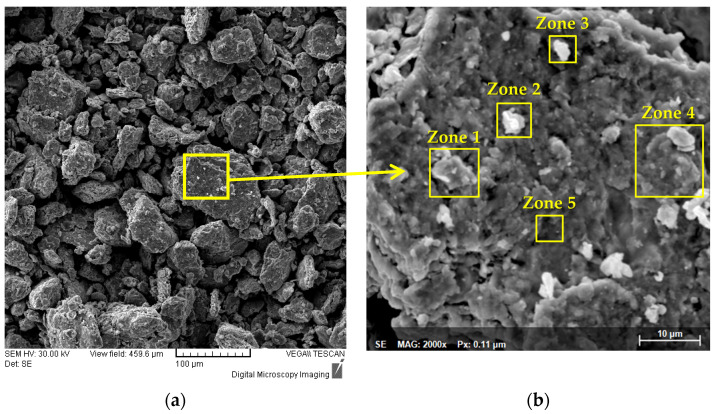
Results of the SEM analyses for (**a**) a CoCrMoNbTi HEA metallic powder sample and (**b**) an agglomerated CoCrMoNbTi HEA particle.

Figure 5 presents results of the analyses of the 30 h alloyed sample at different magnifications that enables to observe its morphology. From Figure 5a, it can be observed that on a particle approx. 100 µm in dia. all the constituent elements of the high-entropy alloy are present as a result of the continuous welding and fracturing that occurs during the mechanical alloying processing. By correlating the mapping analyses with the results of the SEM analyses, particle fractions were fractured and welded to the main particle analyzed in Figure 5a. In Figure 5b, five zones were highlighted and analyzed by size and chemical composition criteria.

In Zone 1, an agglomerated particle with a dia. of roughly 7.5 µm presented Co, Ti and Nb in abundance in its composition. Zone 2 presents a particle composed of Mo, Nb, Cr and Ti in abundance and Zone 3 highlights a particle with Nb abundance both with an approx. dia. of 4 µm. Zone 4 presents a particle with a dia. of 12 µm mainly composed of Mo, Nb and Co, while Zone 5 presents a particle mainly composed of Cr with an approx. dia. size of 2.7 µm.

By comparing the results with the mean particle size of the elemental materials presented in the Section 2, a significant size reduction due to the alloying process can be observed. The particles present a desired composition for this case, where the alloy was confirmed, but it can also be observed that the mechanical alloying took place at a nanoscale, according to Figure 4. According to the analysis of intermediary samples, the 30 h alloying time was considered sufficient for this experimental stage.

**Figure 6:**
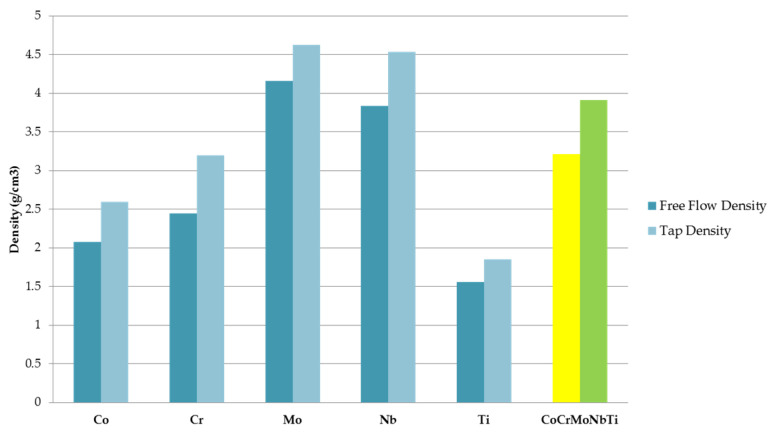
Free flow density and tap density comparison for the raw elemental powders and the obtained CoCrMoNbTi high-entropy alloy after 30 h of milling.

The alloyed metallic powder was technologically characterized in order to establish further possible processing methods. From the free flow density and tap density measured on the elemental pure powder materials and the obtained high-entropy alloys, an improvement could be observed when compared with the elemental materials. The highest values were obtained for Mo, with a free flow density of 4.16 g/cm^3^ and a tap density of 4.63 g/cm^3^. The lowest values were measured for Ti, with a free flow density of 1.56 g/cm^3^ and a tap density of 1.85 g/cm^3^. For the CoCrMoNbTi HEA, a free flow density value of 3.21 g/cm^3^ and tap density value of 3.91 g/cm^3^ were measured. Calculating the ratio between the free flow density and the tap density resulted in the packing density for the CoCrMoNbTi HEA being 82.1%, indicating the possibility of further consolidation of the sample via pressing and sintering. The results are presented graphically in Figure 6.

**Figure 7:**
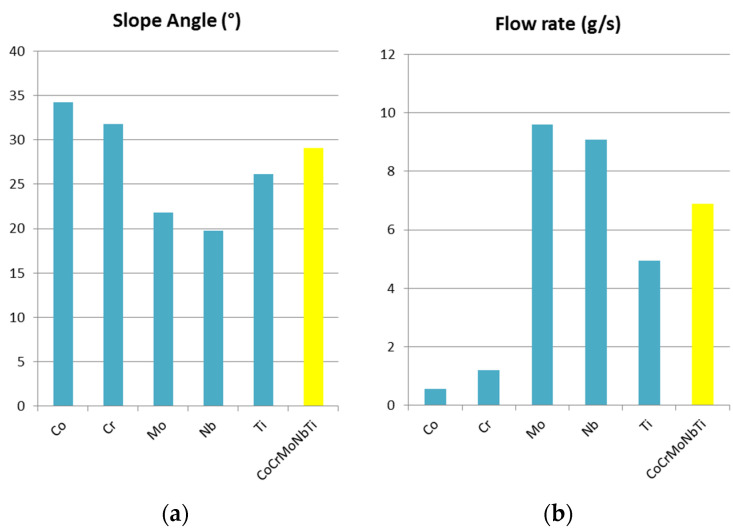
Comparison of the (**a**) slope angle and (**b**) flow rate between the elemental metallic powder and CoCrMoNbTi high-entropy alloy.

The flow rate and slope angle measurements are presented in Figure 7. Similar to the density analyses, a comparison between the raw metallic powder materials and the final alloy was performed. From the flow rate analyses (Figure 7b), a value of 6.89 g/s was measured for the studied HEA, which indicated that the metallic powder could be further processed into coatings with thermal spraying. By alloying, the overall flow rate was improved for the final alloy in comparison with the individual elements. The slope angle (Figure 7a) value of 29.1 degrees also indicated a good flow and a good die filling when pressing the powder.

**Figure 8:**
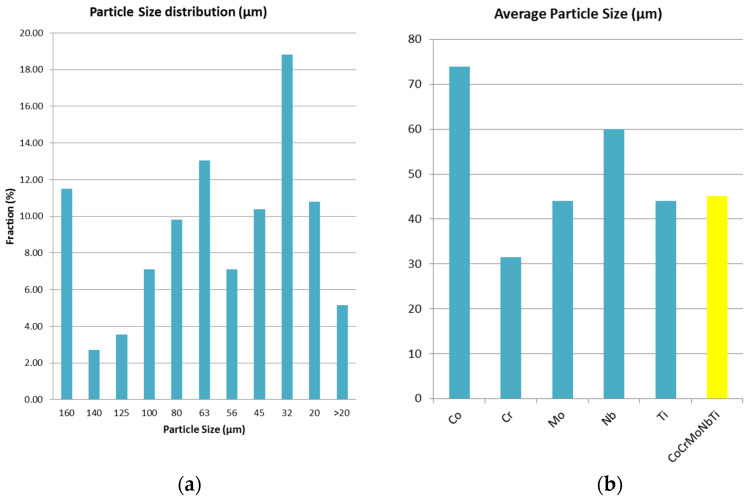
Technological characterization of (**a**) particle size distribution of the obtained HEA and (**b**) average particle size comparison between the elemental materials (initial metallic powders) and the obtained HEA.

In Figure 8a, the particle size distribution for the CoCrMoNbTi HEA milled for 30 h is presented. In Figure 8b, the average particle sizes for the elemental materials and CoCrMoNbTi HEA are presented. It was observed from the particle size distribution graphic that the highest fraction of 18.82% was obtained after 30 h of milling for particles with a diameter between 45 µm and 32 µm, which, compared with the average particle size of the elemental materials, results in a particle dimension reduction. Due to the welding and breaking of the particles during the process, 11.5% of particles were found to be larger than 160 µm in diameter, possibly due to the ductility of titanium and with this being confirmed with the SEM analyses. Overall, the average particle size for the CoCrMoNbTi high-entropy alloy was determined and a value of 45.12 µm was found, indicating a size reduction of the raw Co and Nb metallic powders during processing.

A value of 6 was obtained for CoCrMoNbTi HEA from the VEC calculations, indicating the formation of the BCC phase in the alloy, with the possibility of FCC minor phases being present.

**Figure 9:**
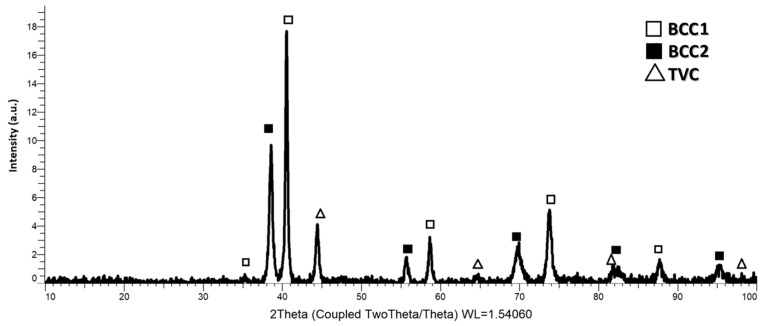
Results of the XRD analyses for high-entropy alloy metallic powder sample with crystallographic phases and their angular positions identified.

X-ray diffraction analyses were carried out for the sample collected from the alloy milled for 30 h in order to validate the theory according to which the material should have a majority of BCC phases, and the results are presented in Figure 9. The crystallographic phases were identified following the XRD analyses performed on the sample in the form of high-entropy alloy metallic powder. After 30 h of mechanical alloying, dual BCC1 and BCC2 phases were identified, along with the TVC phases. The highest peak intensity observed was at 2θ 40.1°, which was preferentially oriented on the (110) plane, with molybdenum abundance as the BCC1 phase. This crystalline phase was also identified at 2θ 58°, 73° and 87°.

According to the recent literature [37], when mixing the elemental powders in equiatomic ratios of the CoCrMoNbTi HEA for 6 h and using it as a coating deposited with the laser cladding technique, the main diffraction peak (namely the solid solution based on BCC phases preferentially oriented on the (110) plan) with dual BCC1 and BCC2 phases is identified after 6 h of mixing.

After 12 h of alloying of the equiatomic high-entropy alloy, Longjun He et.al. [38] identified two BCC phases (BCC1 and BCC2) with different lattice constraints at approx. 2θ 41° and 41.5° angular positions, respectively. Both cases [37,38] obtained the high-entropy alloys as matrices which were reinforced with ceramic particles in order to further produce a coating with laser cladding. This potentially indicates that when the alloying time is increased, the material could also form a solid solution based on the TVC, as identified in this study. At 2θ 38°, 55°, 69°, 82° and 94°, a BCC2 phase with an abundance of niobium was observed. At 2θ 35° and 41° angular positions, a BCC1 phase with an abundance of titanium and 44.6°, 65° and 82.5° angular positions presented the Co0.86Nb0.14 compound with a BCC2 phase. The TVC phase with a Chromium-like crystal structure was identified at 2θ 44.5°, 65° and 98°. The identified tetragonal phases indicate the possible presence of both FCC and BCC phases.

When the CoCrMoNbTi HEA was obtained with vacuum arc melting [39], with variable Ti content (molar ratio 0.5 and 1.0), a high intensity BCC solid solution peak at 2θ of approx. 40° was identified along with the Cr_2_Nb Laves phases at 2θ of approx. 36° and 44°. Nb was present in similar 2θ positions when comparing the results, but when mechanically alloyed, a tendency of forming compounds with Co was observed.

The theory was confirmed following the analysis of the obtained results, whereby the value of 6 obtained from the calculation of the valence electron concentration indicated an abundance of the BCC phases (dual BCC1 and BCC2 phases), but with the value being close to the range, namely 6.8, there was a possibility for the formation of FCC phases, which were also confirmed from the tetragonal phases in chromium-rich areas.

## 4. Conclusions

The alloying degree evaluation was obtained by microstructure analyses, which revealed that the optimal milling time was obtained at 30 h, with a high alloying degree and no contamination.

The results of the mapping analyses presented a good distribution of the elemental metallic powders. Furthermore, when analyzing a singular particle, the constituent components were highlighted.

The results of the SEM analyses presented particles with an increased uniformity in size and shape, where the elemental materials were agglomerated and were not separated. However, a particle size reduction was also observed, having an average dia. of 45 µm for the CoCrMoNbTi HEA according to the technological characterization.

The results of the XRD analyses confirmed the theory and indicated that the dual BCC1 and BCC2 phases with the highest intensity were identified as a BCC1 structure, and were preferentially oriented on the (110) plan and minor tetragonal structures, which can be expected with a high ductility and fracture strength of the final alloy. The TVC phase was identified along the dual BCC phases, indicating the possibility of FCC phases being present in the structure and increased ductility of the studied material.

Our future work will be directed toward the metallic powder consolidation in order to mechanically test the bulk of the final material, but also to obtain electrospark deposition electrodes, which can be obtained with the 82.1% packing density calculated after measuring the free flow and tap density.

## Figures and Tables

**Table 1:** Nominal composition of the elemental powders (wt%) used to obtain the CoCrFeNbTi high-entropy alloy.

CoCrMoNbTi	Co	Cr	Mo	Nb	Ti
Element [at%]	20	20	20	20	20
Element [wt%]	16.95	14.96	27.60	26.73	13.77

## Data Availability

Not applicable.
